# The potential renal toxicity of silver nanoparticles after repeated oral exposure and its underlying mechanisms

**DOI:** 10.1186/s12882-021-02428-5

**Published:** 2021-06-18

**Authors:** Hamed Nosrati, Manijeh Hamzepoor, Maryam Sohrabi, Massoud Saidijam, Mohammad Javad Assari, Nooshin Shabab, Zahra Gholami Mahmoudian, Zohreh Alizadeh

**Affiliations:** 1 Department of Anatomical Sciences, School of Medicine, Hamadan University of Medical Sciences, Hamadan, Iran; 2 Research Center for Molecular Medicine, Hamadan University of Medical Sciences, Hamadan, Iran; 3 Research Center for Health Sciences, School of Public Health, Hamadan University of Medical Sciences, Hamadan, Iran; 4 Endometrium and Endometriosis Research Center, Hamadan University of Medical Sciences, Hamadan, Iran; 5 Department of Anatomical Sciences, Hamadan University of Medical Sciences, Shahid Fahmideh Ave., P.O. Box. 65178-518, Hamadan, Iran

**Keywords:** Silver nanoparticles, Toxicity, Apoptosis, Kidney

## Abstract

**Background:**

Silver nanoparticles (AgNPs) can accumulate in various organs after oral exposure. The main objective of the current study is to evaluate the renal toxicity induced by AgNPs after repeated oral exposure and to determine the relevant molecular mechanisms.

**Methods:**

In this study, 40 male Wistar rats were treated with solutions containing 30, 125, 300, and 700 mg/kg of AgNPs. After 28 days of exposure, histopathological changes were assessed using hematoxylin-eosin (H&E), Masson’s trichrome, and periodic acid-Schiff (PAS) staining. Apoptosis was quantified by TUNEL and immunohistochemistry of caspase-3, and the level of expression of the mRNAs of growth factors was determined using RT-PCR.

**Results:**

Histopathologic examination revealed degenerative changes in the glomeruli, loss of tubular architecture, loss of brush border, and interrupted tubular basal laminae. These changes were more noticeable in groups treated with 30 and 125 mg/kg. The collagen intensity increased in the group treated with 30 mg/kg in both the cortex and the medulla. Apoptosis was much more evident in middle-dose groups (i.e., 125 and 300 mg/kg). The results of RT-PCR indicated that Bcl-2 and Bax mRNAs upregulated in the treated groups (*p* < 0.05). Moreover, the data related to EGF, TNF-α, and TGF-β1 revealed that AgNPs induced significant changes in gene expression in the groups treated with 30 and 700 mg/kg compared to the control group.

**Conclusion:**

Our observations showed that AgNPs played a critical role in *in vivo* renal toxicity.

## Introduction

Advances in nanotechnology have greatly enhanced its potential usage in domestic, industrial, and biomedical applications [1, 2]. Due to their unique physicochemical and biological properties, AgNPs are widely used, indicated by a significant increase in the number of products that contain AgNPs from about 30 in 2006 to more than 435 in 2015 [3, 4]. AgNPs have also been widely used in biomedical applications, such as wound healing, drug delivery systems, catheter modification, dental applications, and bone tissue engineering [5]. According to PubMed statistics, the number of studies on “silver nanoparticles” has also increased over the last decade (i.e., from 896 papers in 2010 to 2529 in 2020). Because of their small size, AgNPs can enter the human body through ingestion [6], inhalation [7], and skin contact [8]. Kidneys, lungs, nervous system, and liver are organs prone to the accumulation of AgNPs [7, 9]. Induction of inflammatory and cytotoxicity effects by AgNPs in different cells and tissues has been reported in studies performed *in vitro* and *in vivo* [9–11]. The most well-known mechanism of AgNPs cytotoxicity involves the induction of oxidative stress, caused by the generation of intracellular reactive oxygen species (ROS), glutathione depletion, a decrease in superoxide dismutase enzyme activity, and an increase in lipid peroxidation [12]. It has been reported that the oxidative stress induced by AgNPs is independent of the toxicity of Ag^+^ ions generated within the cells [13].

Decreased renal function due to the apoptosis of intrinsic renal cell populations is a characteristic feature of renal disorders, caused by different etiologies, such as toxins. Enhanced apoptosis has been shown to lead to glomerulosclerosis and tubular atrophy [14].

The induction or suppression of apoptosis are influenced by a large number of growth factors. For instance, the epidermal growth factor (EGF) has an *in vitro* protective effect on renal cells against apoptotic stimuli, such as serum deprivation [15]. In contrast, it has been reported that the transforming growth factor-β1 (TGF-β1) and the tumor necrosis factor-α (TNF-α) induce apoptosis in renal fibroblasts, tubular epithelial cells [16, 17], and glomerular epithelial cells [18].

The main objective of the current study is to evaluate the extent of renal toxicity induced by different doses of AgNPs after 28 days of oral exposure and to determine the relevant molecular mechanisms. To do so, we assess the effects of AgNPs on the kidney in terms of (1) the reno-somatic index, (2) serum creatinine and blood urea nitrogen levels, (3) histological changes, and (4) changes in the expression of apoptosis-associated and growth factors genes.

## Material and methods

### Animals

In a controlled environment of an animal house (a temperature of 21 ± 2 °C, a humidity of 50 ± 15%, and a light/dark cycle of 12 h), forty 10 to 12-week old adult male Wistar rats (180–200 g) were housed during the experimental period (28 days) with access to water and food ad libitum. The animal bodyweights were recorded before and after the treatment with AgNPs. The bodyweight gain was calculated by subtracting the initial body weight from the final bodyweight [9]. The reno-somatic index (RSI) was calculated according to the following standard formula:

$$ \mathrm{RSI}=\mathrm{Kidney}\ \mathrm{weight}\ \left(\mathrm{g}\right)/\mathrm{bodyweight}\ \left(\mathrm{g}\right)\times 100 $$

### Silver nanoparticles

The suspension of the AgNPs powder (US Research Nanomaterials Inc., Houston, TX, USA, Stock#: US1008) was prepared in accordance with the procedure described in our previous study [19]. Briefly, different concentrations of AgNPs (i.e., 30, 125, 300, and 700 mg) were dispersed in deionized water by vortexing, followed by sonication for 10 min. The particle-size distribution of AgNPs was measured using the dynamic light-scattering (DLS) technique by a Malvern Zetasizer (Nano ZS ZEN-3600, UK). Moreover, a transmission electron microscope (TEM) (Philips-EM 208) was used to determine the size and shape of the nanoparticles.

### The experimental design

The animals were randomly divided into four treatment groups and a control group (*n* = 8 for each group). Rats in the first four groups were administered 30, 125, 300, and 700 mg/kg of AgNPs suspension orally for 28 days, respectively. Equal volumes of deionized water were administered to the control group [11]. The animals were sacrificed 24 h after the last administration to harvest and weigh their kidneys. The left kidney of each rat was frozen at −80 °C for molecular studies, while the right kidney was immersed in a 10% neutral buffered formalin solution for further histopathological investigations.

### Determination of blood urea nitrogen (BUN) and creatinine

Blood samples were collected through a cardiac puncture and allowed to clot for 45 min at room temperature. The serum was separated by centrifugation at 1500 g for 10 min. BUN and creatinine were measured using an AutoAnalyzer (Hitachi 7180, Hitachi, Japan) and standard assay kits.

### The histological study

The fixed tissues were processed routinely using the standard technique, followed by being embedded in paraffin, and 5 μm-thick sections were prepared for hematoxylin and eosin (H&E) staining. Special staining techniques, Masson’s trichrome, and periodic acid-Schiff (PAS) were performed to evaluate the collagen deposition changes, brush border, and tubular basal lamina, respectively. All the images were acquired by a Moticam 2000 camera (Kowloon, Hong Kong) attached to a Nikon Eclipse E800 research transmitted light microscope (Melville, New York, USA). The Motic Images 2.0 software was used for H&E and PAS stained sections and ImageJ (version 1.52a) was used for Masson’s trichrome stained slides to evaluate the variations in the histomorphology of the groups.

### Terminal deoxynucleotidyl transferase dUTP nick end labeling (TUNEL) assay

A TUNEL assay kit (Promega Co., Madison, WI, USA, CAT. #G7130) was used to assess cell apoptosis in renal tissues according to the instructions provided by the manufacturer. Paraffin-embedded tissue sections were deparaffinized, rehydrated, and permeabilized with a proteinase K solution for 30 min. Tissue sections were then incubated with biotinylated nucleotide mix, recombinant terminal deoxynu-cleotidyl transferase (TdT), and an equilibration buffer for 1 h at 37 °C. The sections were incubated in a converter-POD solution for 30 min after rinsing with PBS. Dehydration was performed using graded ethanol. The sections were covered with the mounting medium (Scytek Laboratories, Logan, UT, USA) and counterstained with Mayer’s Hematoxylin Solution. During the tailing reactions, TdT was eliminated as a negative staining control.

The average number of apoptotic cells was determined by counting TUNEL-positive cells in five neighboring fields at a magnification of ×400, dividing the total by five, and expressing the result as a percentage.

### Immunohistochemistry

Changes in the distribution and expression of the caspase-3 protein in renal tissues were evaluated by immunohistochemistry in the prepared kidney sections. Briefly, tissue sections with a thickness of 4 μm were sequentially deparaffinized, rehydrated, and submitted for antigen retrieval. The sections were incubated overnight at 4 °C with the rabbit anti caspase-3 antibody (ab13847, 1:200) diluted in PBS. After washing, the secondary antibody bond to the biotin (Detection Kit Goat Anti-Rabbit HRP (IgG) (Ab6721) Abcam) was applied for 15 min, and then streptavidin-horseradish peroxidase (HRP) was added, followed by incubation in a moisture box at room temperature for 15 min. The results were developed using DAB, and the sections were mounted and observed under a light microscope. Immuno-positive cells were counted and expressed as percentage.

### RNA extraction and quantitative realtime polymerase chain reaction

Total RNA was extracted from the renal tissues of the rats using the TRIzol® reagent (Invitrogen), according to the protocol provided by the manufacturer. The RNA concentration was determined using an Epoch Microplate Spectrophotometer (Biotek, USA). The extracted RNA was reverse transcribed into single-strand cDNA using the RevertAid™ first-strand cDNA synthesis kit (Thermo Scientific) according to the manufacturer’s instructions. The transcription process included incubation of the reaction mixture at 20 °C for 30 s, followed by 5 min at 44 °C, 30 s at 55 °C, and 5 min at 95 °C. The cDNA was stored at −80 °C until further use for PCR.

Quantitative real-time PCR analyses were carried out using the SYBR premix Ex Taq 2 kit (Takara) in a final volume of 25 μl with 10 pmol of each primer by the CFX96 real-time PCR detection system (BioRad, USA). Each assay was run in the triplicate manner with each set of primers. The sequences of primers, accession number, and primer-specific annealing temperature are presented in Table 1.

**Table 1:** Features of the primers of the studied genes

Gene	Accession number	Primer sequence	Annealing temperature
Bax	NM_017059	F: GAGACACCTGAGCTGACCTTG R:CCTGCCACACGGAAGAAGACCTC	55
Bcl-2	NM_016993	F: CGGGAGAACAGGGTATGATA R: TCAGGCTGGAAGGAGAAGATGC	54
EGF	NM_012842	F: AACTGTGTCATTCCAGGATC R:CGAGTCCTGTAGGATCGCCAT	55
TGF-β1	NM_021578.2	F: ATTCAAGTCAACTGTGGAGCAAC R: CGAAAGCCCTGTATTCCGTCT	57
TNF-α	NM_012675.3	F: TGTTCATCCGTTCTCTACCCA R: CACTACTTCAGCGTCTCGT	55
18SrRna	NM_031144	F:CGGAAGACTCACACCTTGA R:GTCCTCAGTGTAGCCCAAGA	53

Cycle threshold (Ct) values were obtained using the auto Ct function. The mean Ct value was determined after efficiency correction, and it was then normalized to the reference gene (18S rRNA) using delta (∆) Ct. The relative expression changes were determined using the 2^−∆∆Ct^ method [20].

### Statistical analysis

The statistical analysis was performed using SPSS 16.0 (IBM, USA), and the data were presented as mean ± standard deviation (SD). The statistically-significant changes were determined using a one-way analysis of variance (one-way ANOVA) and the Tukey test. *P*-values less than 0.05 (*p* < 0.05) were considered statistically-significant changes. The Pearson’s correlation analysis was used to determine the correlation values (r) between the parameters.

### Ethical statement

The above-mentioned treatment/sampling protocols were approved by the Ethics Committee of Hamadan University of Medical Sciences (ethical code: IR.UMSHA.REC.1394.553). Moreover, all the methods were carried out in accordance with the relevant guidelines and regulations. The study was carried out in compliance with the ARRIVE guidelines.

## Results

### Nanoparticles characterization in solution

**Fig. 1:**
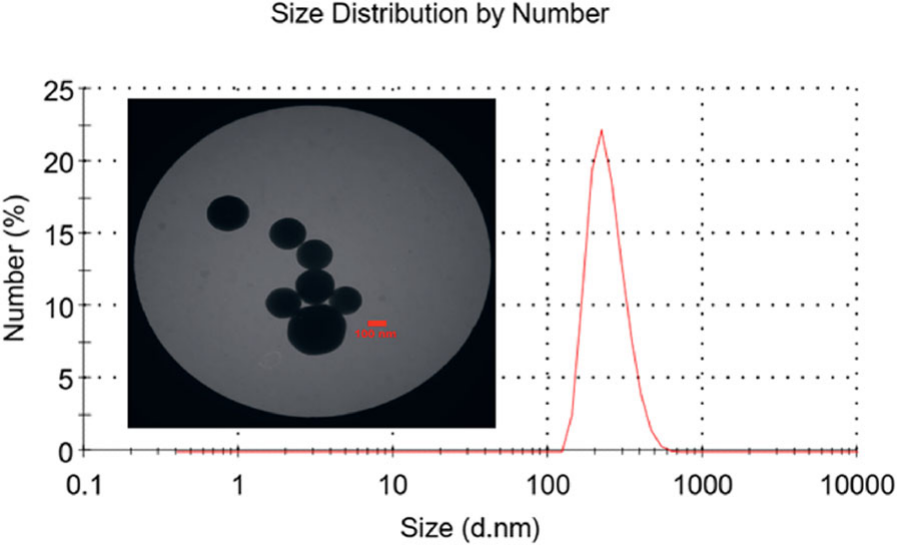
TEM micrograph and particle size distribution of AgNPs

The AgNPs suspension was subjected to dynamic light scattering (DLS) analysis to determine the diameter of the nanoparticles. It exhibited a hydrodynamic diameter peak with an average size of approximately 250 nm. The TEM images of AgNPs showed that the majority were spherically-shaped with smooth surfaces (Fig. 1).

### Body and organ weight

Table 2 shows the results of bodyweight gain and the reno-somatic index. There were no significant dose-related changes in the bodyweight gains of the rats. In addition, no significant changes in the reno-somatic index were observed in the treated rats, except for an increase (*P* < 0.05) in the index of the right kidney for the group receiving 30 mg/kg.

**Table 2:** Bodyweight gain, the reno-somatic index, and the serum levels of BUN and creatinine after 28-day oral administration of AgNPs (mean ± S.D.)

Groups	Body weight gain (g)	Reno-somatic index (left kidney) (%)	Reno-somatic index (right kidney) (%)	BUN (mg/dL)	Creatinine (mg/dL)
Control	72.72 ± 19.50	0.37 ± 0.05	0.35 ± .04	67.60 ± 4.72	0.70 ± 0.07
30 mg/kg	61.02 ± 8.045	0.42 ± 0.02	0.43 ± 0.04*	62.25 ± 3.20	0.70 ± 0.08
125 mg/kg	76.63 ± 18.57	0.37 ± 0.03	0.36 ± 0.04	59.50 ± 1.73	0.68 ± 0.05
300 mg/kg	64.52 ± 7.98	0.35 ± 0.03	0.36 ± 0.02	62.50 ± 5.00	0.78 ± 0.05
700 mg/kg	67.05 ± 2.29	0.36 ± 0.03	0.37 ± 0.02	62.50 ± 3.00	0.60 ± 0.08

*Significant difference compared to the control, *p* < 0.05

### Blood urea nitrogen (BUN) and creatinine

In this study, kidney function was evaluated based on the serum levels of BUN and creatinine. However, these parameters showed no statistically-significant differences between experimental and control groups after 28 days of exposure to AgNPs (Table 2).

### Histological evaluation

#### H&E-stained sections

**Fig. 2:**
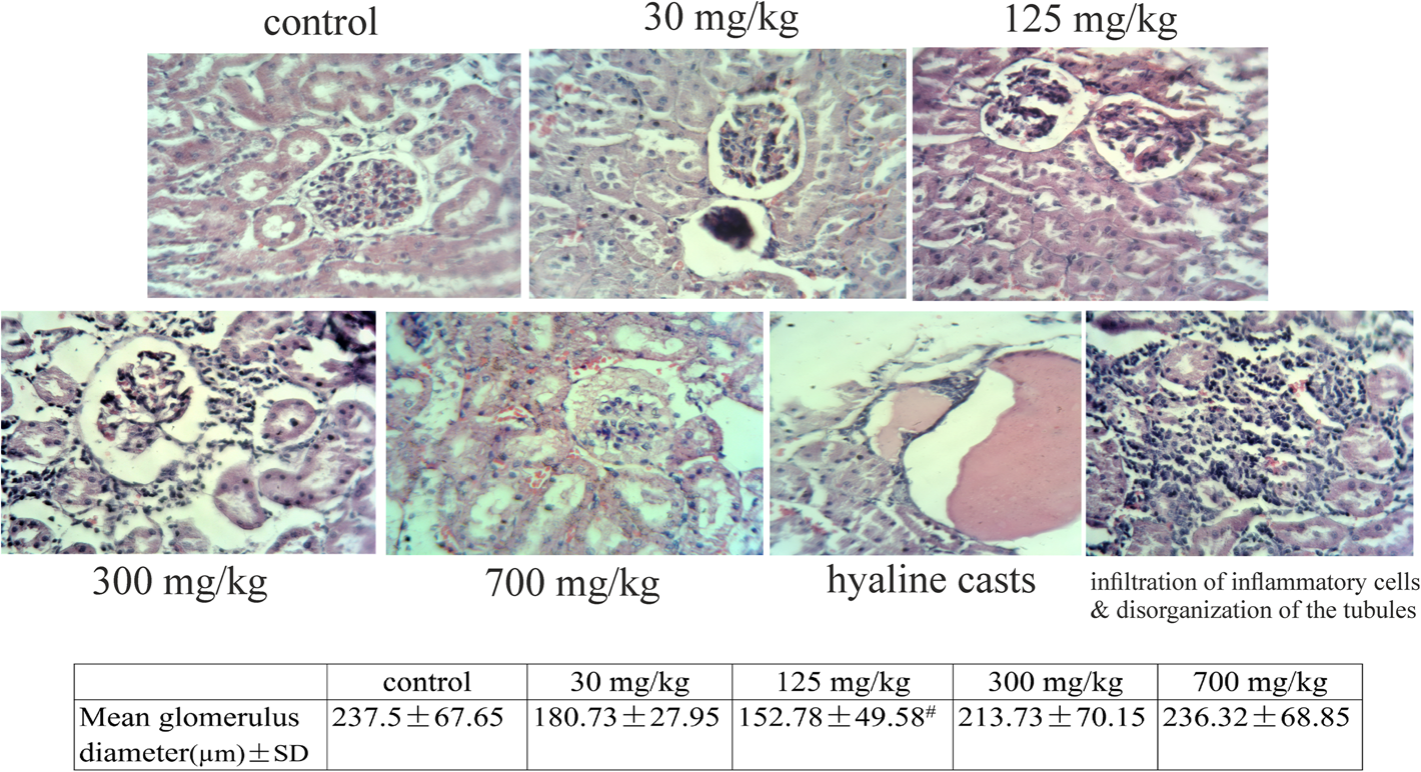
Light micrographs of rat kidney sections (H&E stain, ×400). The renal cortex shows a normal architecture in the control group. Groups treated with 30, 125, and 300 mg/kg show marked degenerative changes in the glomeruli, showing necrosis with loss of glomerular tufts and wide Bowman’s space. The group treated with 700 mg/kg shows swelled renal glomerulus. Hyaline casts in renal tubule are observed in the group treated with 125 mg/kg. Congestion of the capillary loops, infiltration of inflammatory cells, and disorganization of the tubules are seen in the groups treated with AgNPs. The effects of various concentrations of AgNPs on the diameter of the glomeruli are presented and compared to the control group. #*P* < 0.001

Tissue sections from the control group showed a typical histological structure in different parts, such as renal corpuscles, proximal and distal convoluted tubules, and the interstitial tissue. Sections from groups treated with AgNPs showed many forms of glomerular, tubular, and interstitial changes (Fig. 2).

- **Glomerular Alterations**: The cortex showed partial destruction of renal corpuscles with collapsed glomerular tufts, widening of the Bowman’s space, and necrosis. The mean glomerular diameter was also measured. This parameter reduced significantly in the group treated with 125 mg/kg compared to the control group (*p* < 0.05).
- **Tubular Alterations**: In groups treated with AgNPs, loss of tubular architecture was observed, and the epithelial lining of the tubules in the cortex showed cytoplasmic necrosis and vacuolation. The luminar site of several cortical tubules displayed dense acidophilic hyaline casts. Moreover, shedding and desquamation of the lining epithelium were observed more prominently in the proximal tubules of the groups treated with 30 and 125 mg/kg.
- **Interstitial Tissue Alterations**: The most significant histological changes in the renal interstitial tissue, including infiltration of inflammatory cells and congestion, were observed in rats exposed to 125, 300, and 700 mg/kg of AgNPs (Fig. 2).

#### PAS-stained sections

**Fig. 3:**
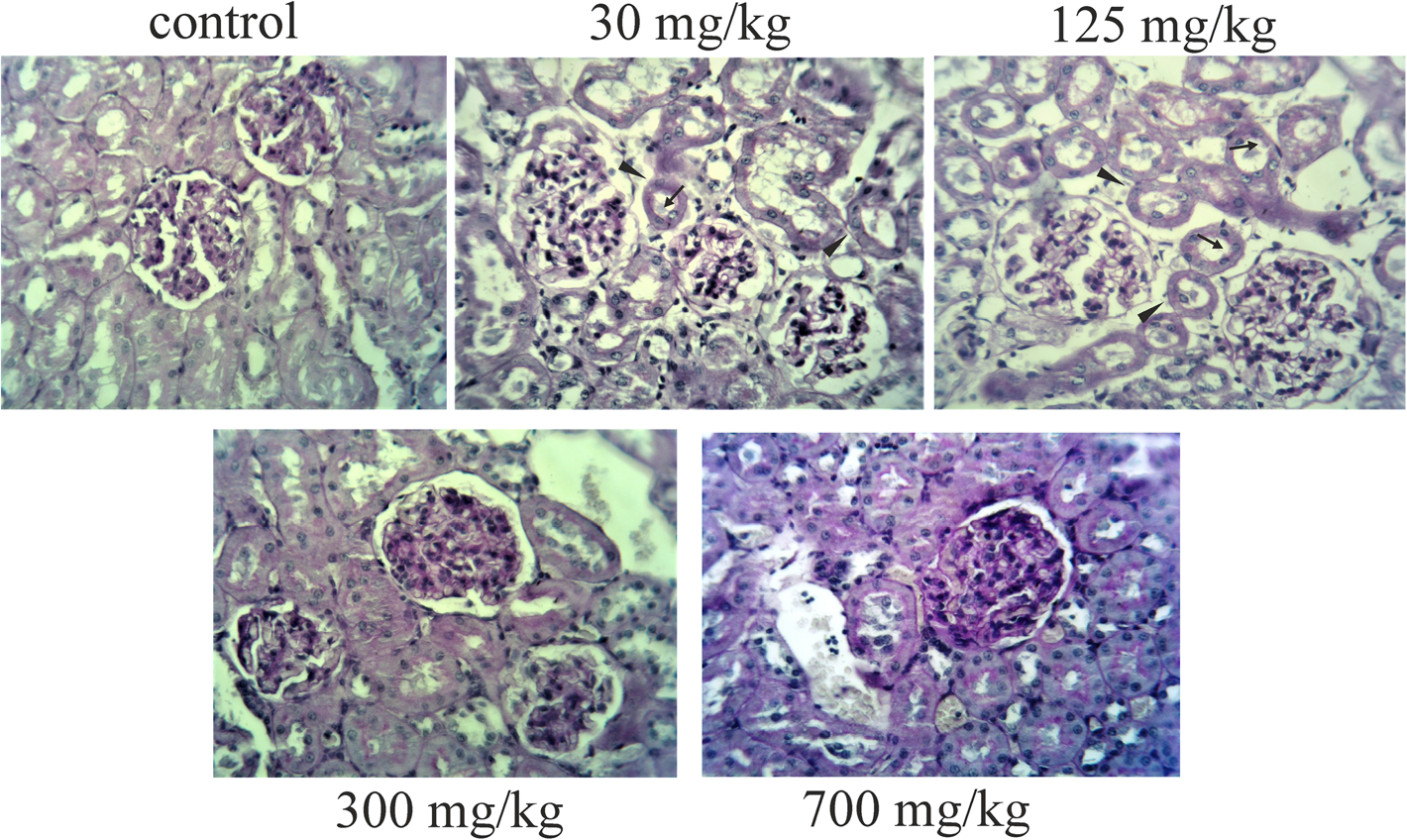
Periodic acid-Schiff (PAS) staining of kidney sections after 28 days of oral exposure to AgNPs (original magnification ×400). The disruption of the brush border (arrows) and the basement membrane integrity (arrowheads) were observed in the renal tubules of the groups treated with 30 and 125 mg/kg of AgNPs

Regarding PAS staining, the disruption of the brush border and the basement membrane integrity were observed in the renal tubules of the groups treated with 30 and 125 mg/kg of AgNPs compared to the control group. The cortical tubules from groups treated with 300 and 700 mg/kg showed a protected brush border and continuous basal lamina (Fig. 3).

#### Masson’s trichrome-stained sections

**Fig. 4:**
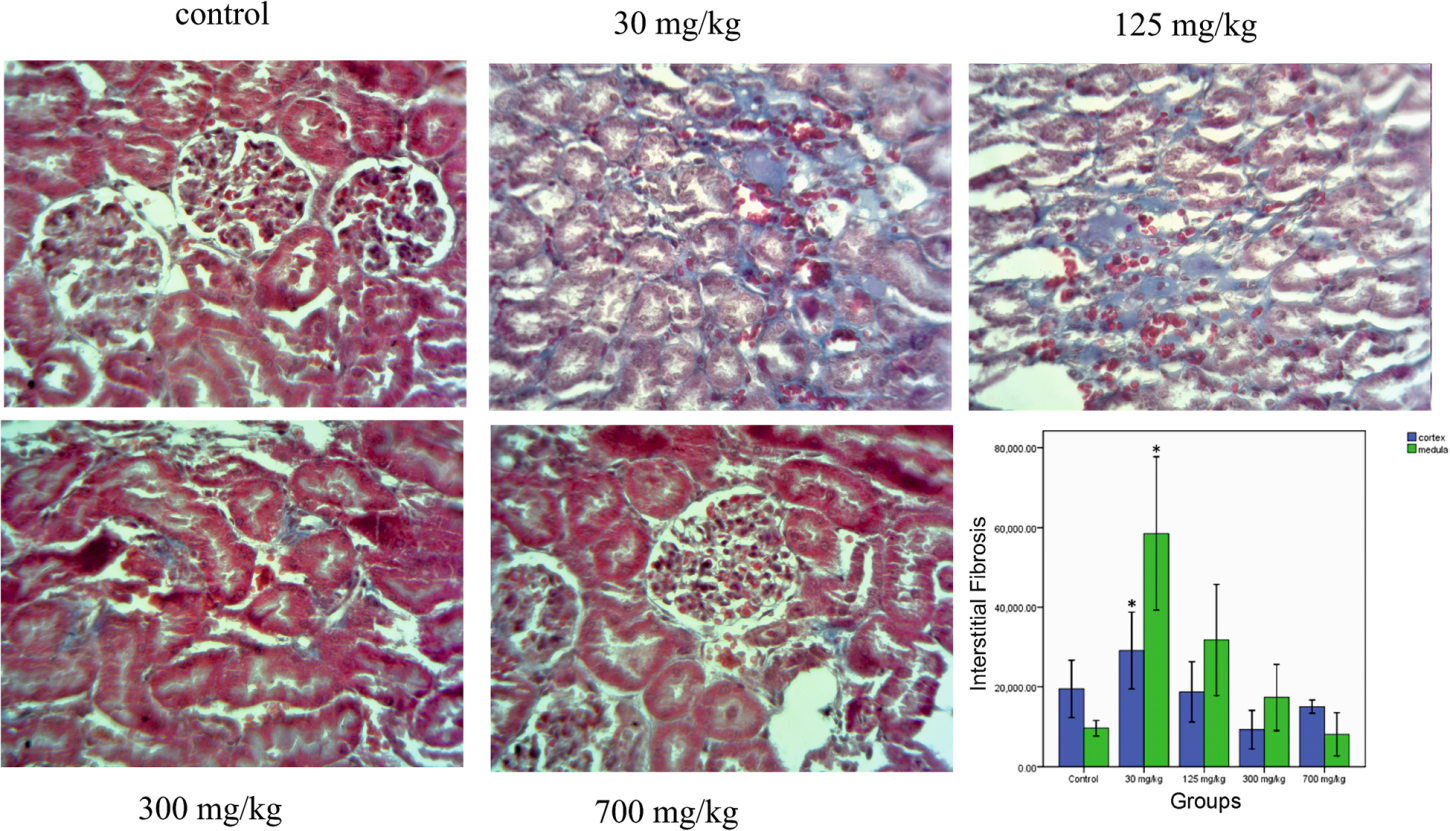
Masson’s trichrome staining (original magnification ×400). Collagen depositions were quantified by counting the number of blue pixels in the medulla and the cortex using the ImageJ software. * indicates a significant difference with the control group (*P* < 0.05)

Masson’s trichrome quantified the blue intensity (representing collagen deposition) within the renal tissue. The blue intensity increased in the groups treated with 30 (*p* < 0.05) and 125 (*p* > 0.05) mg/kg in both the cortex and the medulla. It was shown that in the groups treated with 300 and 700 mg/kg, the collagen content was almost similar in normal renal tissues (Fig. 4). PAS staining confirmed the results of the Masson’s trichrome staining for collagen sedimentation.

### TUNEL assay

**Fig. 5:**
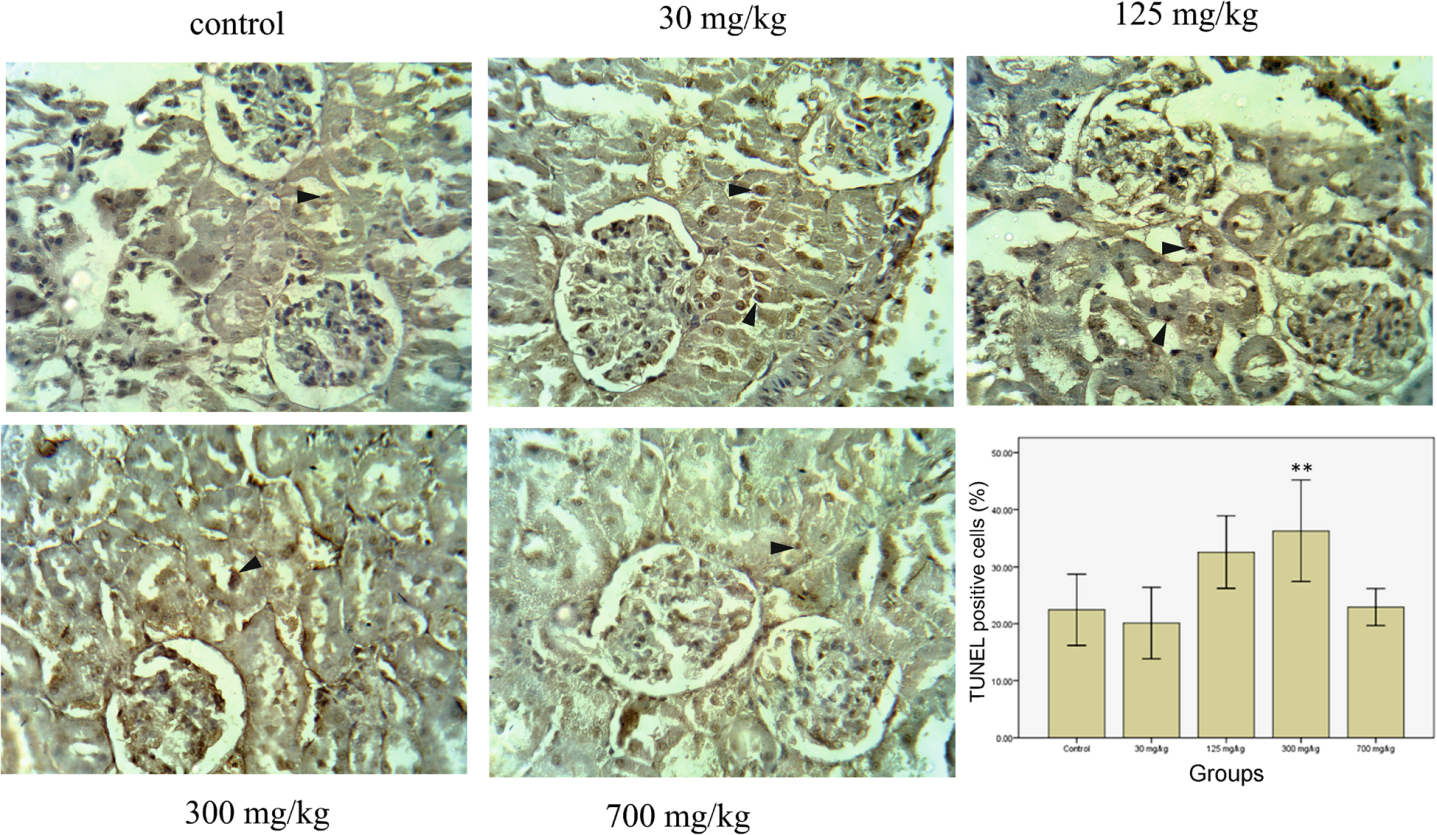
Cell apoptosis (arrowheads) detected by TUNEL in the renal tubule epithelial cells (original magnification × 400). ** indicates a significant difference with the control group (*P* < 0.01)

To assess the effects of treatment with AgNPs on renal tubular cell apoptosis, renal tissue sections were examined by performing TUNEL staining. As illustrated in Fig. 5, the number of TUNEL-positive tubular cells increased in the groups treated with 125 (*p* > 0.05) and 300 (*P* < 0.05) mg/kg compared to the control, 30, and 700 mg/kg groups.

### Immunohistochemistry

**Fig. 6:**
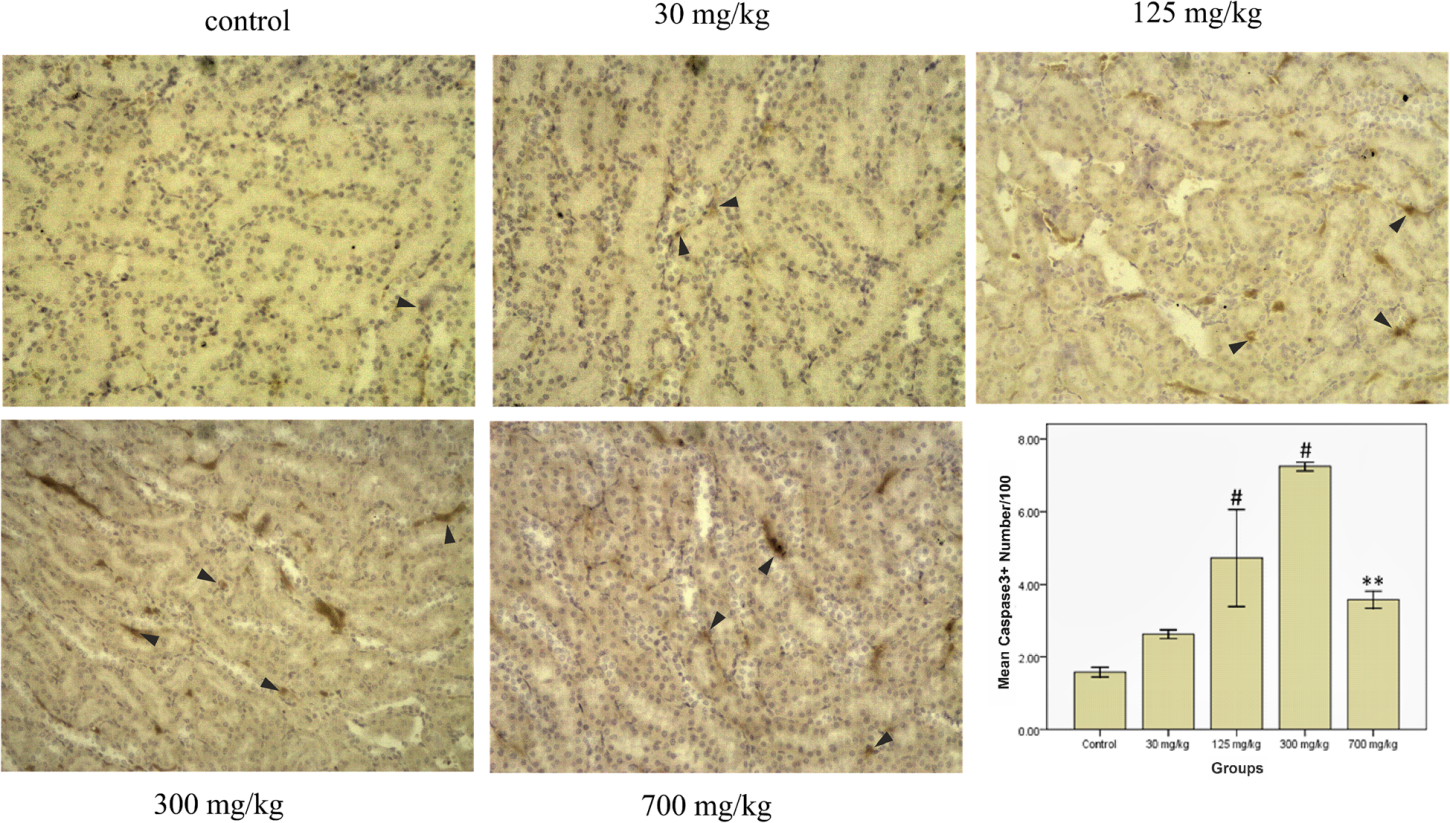
Cell apoptosis detected by caspase-3 immunostaining (arrowheads) in the renal tubule epithelial cells (original magnification ×400). **P < 0.01 and #*P* < 0.001 compared to the control group

The percentages of caspase-3 positive cells were obtained in the stained slides. The results are shown in Fig. 6, indicating a significant difference in the expression of caspase-3 between the groups treated with 125, 300, and 700 mg/kg and the control group (*P* < 0.05).

### Expression of apoptosis-associated genes

**Fig. 7:**
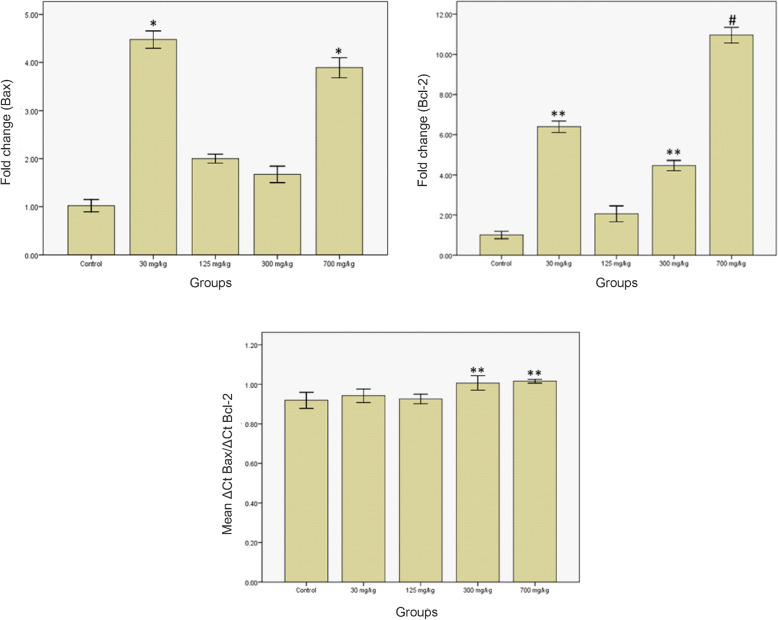
Bax and Bcl-2 genes expression and the ratio of Bax to Bcl-2 changes in the kidney of the rats treated with AgNPs. *P < 0.05, **P < 0.01, and #*P* < 0.001 compared to the control group

As illustrated in Fig. 7, Bax mRNA concentration increased in the groups treated with 30 and 700 mg/kg compared to the controls group. While the expression of this gene increased in groups treated with 125 and 300 mg/kg, this increase was not statistically significant (*P* < 0.05). The expression of Bax in groups treated with 30, 125, 300, and 700 mg/kg was 4.47, 1.91, 1.69, and 3.96 times that of the control group, respectively.

Bcl-2 mRNA concentration increased significantly in groups treated with 30, 300, and 700 mg/kg compared to the control group (*P* < 0.05) (Fig. 7). The average level of Bcl-2 gene expression in groups treated with 30, 125, 300, and 700 mg/kg increased by 6.37, 2.16, 4.42, and 11.69 folds, respectively.

The Bax/Bcl-2 ratios of the mRNA levels are shown in Fig. 7. These ratios significantly increased in the groups treated with 300 and 700 mg/kg compared to the control group.

### Expression of growth factors

**Fig. 8:**
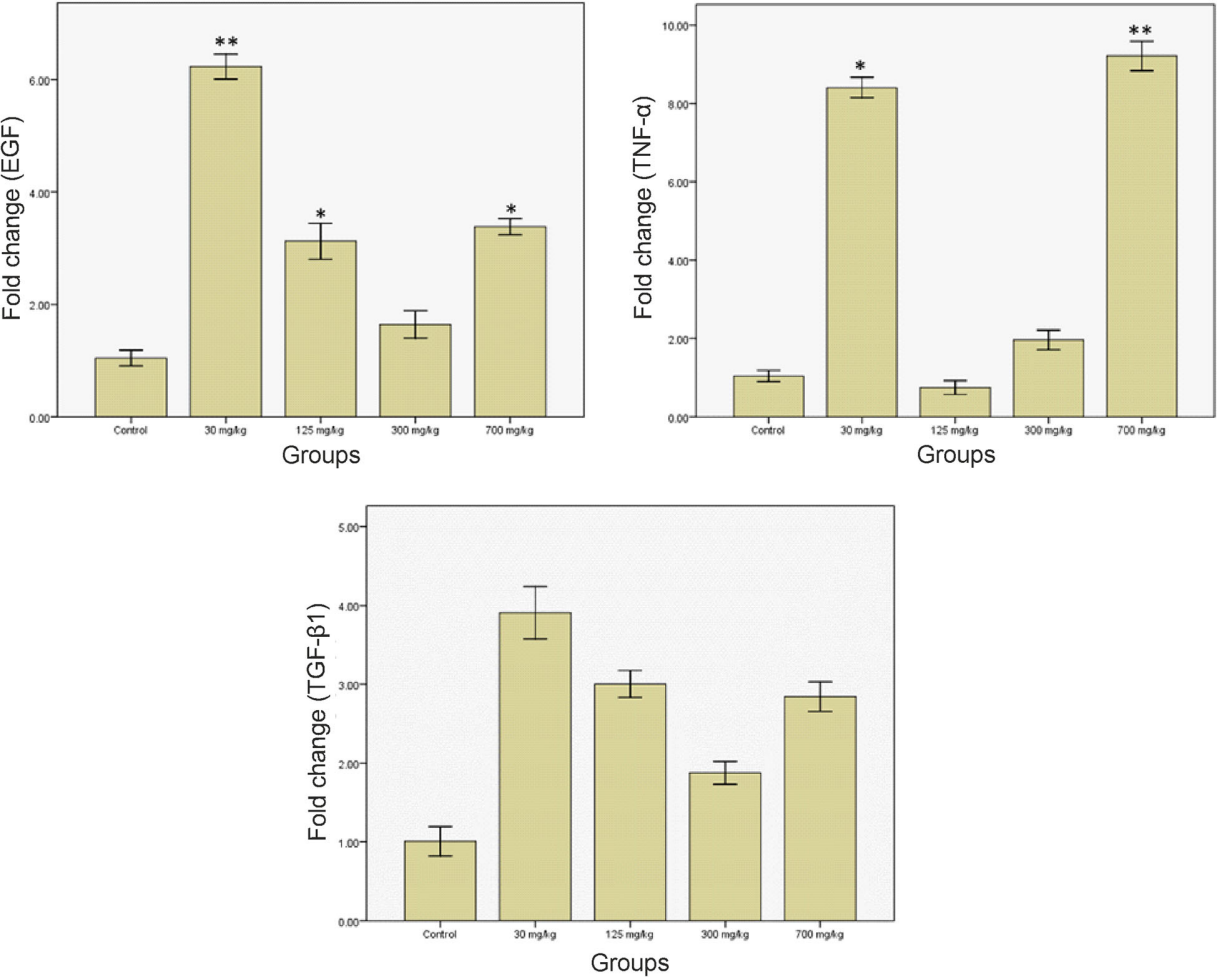
Gene expression of EGF, TNF-α, and TGF-β1. *P < 0.05 and **P < 0.01 compared to the control group

Analysis of the expression of growth factors revealed the upregulation of the EGF mRNA concentration in groups treated with 30, 125, and 700 mg/kg compared to the control group (Fig. 8). Moreover, TNF-α mRNA expression increased in the groups treated with 30, 125, 300, and 700 mg/kg. The results obtained from evaluating TGF-β1 mRNA expression showed no statistically-significant differences between the control group and the experimental groups despite the fact that the increased expression of this gene was seen in groups treated with 30 and 700 mg/kg (*P* > 0.05).

### Correlation between apoptosis and the expression of genes influencing cell death

A significant positive correlation was observed between the mRNA levels of Bax and Bcl-2 (r = 0.850, *P* = 0.0001). The caspase-3 expression was correlated with the ratio of ΔCt Bax/ΔCt Bcl-2 (r = 0.477, *p* = 0.03) and TUNEL-positive cells (r = 0.547, *p* = 0.01). The correlations between TGF-β1, TNF-α, and EGF mRNA expression and transcription levels of Bax and Bcl-2 are shown in Table 3.

**Table 3:** The correlation between apoptosis-related genes and the expression of growth factors in rats treated with AgNPs

	ΔCt Bax	ΔCt Bcl-2
**ΔCt TGF-β1**	r = 0.802 (*p* = 0.001)	r = 0.636 (*p* = 0.003)
**ΔCt TNF-α**	r = 0.610 (*p* = 0.006)	r = 0.749 (*p* = 0.001)
**ΔCt EGF**	r = 0.895 (*p* = 0.001)	r = 0.674 (*p* = 0.002)

## Discussion

Even though AgNPs have numerous advantages that make them ideal for novel biomedical applications, their toxicity has recently become a subject of research [5]. In this regard, *in vitro* studies have revealed nanosilver-related toxic effects in rat neuronal cells and hepatocytes, murine stem cells, and human lung epithelial cells [21, 22].

Exposure to AgNPs can occur in different ways, including dermal contact, inhalation, and ingestion [23, 24]. The oral route, as an exposure route for AgNPs, may be important in many industries, and in food and medicine products. The daily amount of silver derived from natural sources in food and water ingested by humans is approximately 0.5–30 μg [25]. Exposure to AgNPs leads to the translocation of particles to the blood, and their distribution throughout various organs, particularly the kidneys, liver, spleen, brain, and lungs [26]. Kidneys are known as one of the most vulnerable organs after prolonged exposure to nanoparticles [9]. Kim et al. showed that AgNPs accumulated in the kidney after oral administration for 28–90 days [9]. Moreover, the deposition of nanoparticles and silver can occur along the mesangium and glomerular basement membrane [27, 28]. That is why the present study was conducted to study the adverse effects of AgNPs on rat kidney treated with repeated oral administration for 28 days by examining the bodyweight, the reno-somatic index, serum creatinine and blood urea nitrogen levels, histological changes, and changes in the expression of apoptosis-associated genes and growth factors genes.

The results demonstrated that there were no significant dose-related changes in the bodyweight gains of the rats. No significant changes in the reno-somatic index were observed in the treated rats. These results are in line with those of Kim [9] and Ji [7], showing no significant changes in bodyweight and renal index due to various concentrations of AgNPs during the 28-day experiment through oral and inhalation exposure.

BUN and creatinine did not increase significantly compared to the control group; however, inflammatory responses were observed in the kidney. It seemed that the inflammatory responses were too week to impair the filtration capacity of the kidney. Using different doses and durations, other studies have also shown the same results [9, 26, 29].

The histopathological examination of the kidney shows that dose-dependent AgNPs induced lesions in renal corpuscles, tubules, and interstitial tissues, and inflammation. Partial to complete damages to a number of renal corpuscles with loss of glomerular capillary tufts were observed. The morphometry studies confirmed this result by revealing a significant decrease in the diameter of the affected renal corpuscles compared to the control. However, these changes were more prominent in the groups treated with 30 and 125 mg/kg. Marked glomerular capillary-tuft distortion or complete loss have been described in case of severe renal injury and toxic conditions [30–32].

The results related to the histology of the lining tubular epithelial cells showed damage, including vacuolization, cloudy swelling, severe necrosis, pyknotic nuclei, and degenerative changes, together with desquamation of degenerated cells and shedding in the lumen of the tubules. Complete or partial loss of brush border, interrupted basal laminae, and tubular dilatation with intraluminal dense acidophilic hyaline casts were also evident. The results of this study showed that these changes were more frequent in the groups treated with 30 and 125 mg/kg, indicating toxicity induction by these doses. In confirmation of the effects of toxic substances on vacuolar degeneration and cloudy swelling, the evaluations of the effects of cisplatin and different kinds of nanoparticles have shown the same results [33–35]. Almost all observed cytoplasmic and nuclear degenerative changes were more evident in proximal tubules than in distal tubules. This can be because the main primary site of reabsorption and active transmission is the proximal tubules [36].

The hyaline casts represent injured tubular epithelium. The hyaline casts form cellular debris that has undergone molecular changes. Cells and their debris, which is detached from the tubular basement membrane, interact with proteins in the tubular lumen, leading to cast formation. In addition, impaired sodium reabsorption due to the damaged tubular epithelium results in increased sodium concentration in the lumen of tubules, causing protein polymerization and contributing to cast formation [37].

Regarding vascular alterations, consistent with our results, other studies have reported that different nanoparticles with different sizes resulted in expanded and congested renal tubular capillaries with inflammatory infiltration [29, 32]. It has been reported that cell infiltration is a sign of atrophy of tubular cells in chronic kidney diseases [38]. This inflammatory response seems to be a result of the oxidative stress caused by AgNPs, contributing to vascular congestion.

Interstitial tissue fibrosis involves the excessive accumulation of collagen fibrils, and it is a common feature of many diseases that progress to chronic renal failure [39]. Results of this study revealed marked deposition of collagen within the glomeruli and between renal tubules in the groups treated with 30 and 125 mg/kg compared to other groups. In the formation of renal interstitial fibrosis, a variety of inflammatory cells and growth factors, such as TGF-β1, participate. TGF-β1 is considered a key mediator of renal interstitial fibrosis [40]. In our study, the expression of TGF-β1 increased in groups treated with 30 and 125 mg/kg of AgNPs to almost 3.98 and 3 times that of the control group, respectively. Moreover, the mean area percent of collagen fibers in the group treated with 30 mg/kg was significantly higher than that of the control.

In the present study, it seems that the more severe histological changes in rats receiving 30 and 125 mg/kg and the slight damage in groups receiving 300 and 700 mg/kg might be due to the translocation of AgNPs into the kidney. This may be due to the agglomeration of AgNPs used in this study (~250-nm hydrodynamic diameter agglomerates). The small intestine is the first site for the nanoparticles to be absorbed following oral administration. The agglomeration of AgNPs in high concentrations may have also hindered their intestinal absorption, resulting in an insufficient amount of AgNPs being available to the kidney. Kim et al. reported similar results [9].

The number of renal cells during the development and progression of renal disorders is regulated by apoptosis [41]. Through studying the gene expression of apoptotic regulatory and effector molecules in rats treated with different doses of AgNPs, we obtained a greater understanding of controlling apoptosis in the affected kidney.

Bcl-2 is an apoptosis inhibitory factor, while Bax promotes the process of apoptosis in various tissues, and the state of cell apoptosis is determined by the ratio of their levels of expression [42]. We examined whether the increased ratio of these genes was related to the process of apoptosis in the renal tissue of the rats treated with AgNPs. It was found that the number of caspase-3 positive cells significantly increased in the interstitial and tubular epithelial cells of the rats treated with 125 and 300 mg/kg. Furthermore, the ratio of Bax/Bcl-2 mRNA was correlated with caspase-3 positive cells. The findings indicate a possible implication of Bax and Bcl-2 in the apoptotic process during AgNPs treatment.

TUNEL, caspase-3, and Bax/Bcl-2 mRNA evaluation showed a lower rate of apoptosis in the group receiving 30 mg/kg compared to the other tested groups. Given the lack of association between apoptosis (caspase-3 positive cells) and fibrosis or glomerular and tubal injury, alternative non-apoptotic pathways may be activated in this group as more prominent necrosis was found in the group receiving 30 mg/kg.

Activated immune cells produce TNF-α and other pro-inflammatory cytokines in chronic renal disorders, stimulating the release of chemo-attractive molecules by the tubular epithelial cells [43]. It also recruits leukocytes to tubulointerstitium, increasing inflammation, tubulointerstitial damage, and renal dysfunction. Similarly, the results of our study showed that infiltrated immune cells increased in parallel to the increase in the expression of TNF-α mRNAs in the groups receiving 30 and 700 mg/kg. Unlike the group receiving 700 mg/kg, tubulointerstitial damage was observed in the group treated with 30 mg/kg. It seems that the upregulation of TNF-α in the group treated with 700 mg/kg was because of increased infiltrated leukocytes, which indicate the early stage of renal injury. This inflammatory response is because of the oxidative stress caused by AgNPs, resulting in vascular congestion.

The positive association between the expression of TGF-β1 and the severity of the tubulointerstitial injury and renal dysfunction has been reported in various studies. In these studies, the upregulation of TGF-β1 is correlated with an increased risk of progression from chronic renal disease to end-stage renal failure [17, 44].

It has been shown that TGF-β1 contributes to tubulointerstitial damage and renal dysfunction through the loss of tubular epithelial cells by inducing apoptosis and increasing fibrosis. Similarly, our results showed more prominent histological changes in the groups receiving 30 and 125 mg/kg than the group receiving 300 mg/kg.

Our findings revealed an increase in TGF-β1, TNF-α, and EGF mRNA in some treated groups compared to controls; however, they failed to show any statistically-significant correlation between these values and caspase-3 or TUNEL-positive cells.

A study by Gobe et al. has shown that the expression of EGF and Bcl-2 in distal tubules increases in the ischemic kidney, leading to distal tubule stress resistance [45]. On the other hand, according to the significant increase in Bcl-2 in the group receiving 300 mg/kg compared to the other groups, the decreased expression of EGF seems to have led to an increase in apoptosis.

## Conclusions

The present study demonstrated that AgNPs induced renal toxicity at both morphological and molecular levels after repeated oral administration. We conclude that renal fibrosis is associated with progressive tubular and glomerular changes in the experimental groups. Furthermore, the ratio of Bax/Bcl-2 mRNA was correlated with caspase-3 positive cells. The findings indicate a possible implication of Bax and Bcl-2 in the apoptotic process during AgNPs treatment. The transcription levels of TGF-β1, TNF-α, and EGF are correlated with Bax and Bcl-2 mRNA expression, indicating that these growth factors might be involved in the regulation of apoptosis.

## Data Availability

All data generated or analyzed during this study are included in this published article.

## Abbreviations

AgNPs: Silver nanoparticles
BUN: Blood urea nitrogen
Ct: Cycle threshold
DLS: Dynamic light scattering
EGF: Epidermal growth factor
H&E: Hematoxylin and eosin
HRP: Horseradish peroxidase
PAS: Periodic acid-Schiff
RT-PCR: Reverse transcription polymerase chain reaction
TGF-β1: Transforming growth factor β1
TNF-α: Tumour necrosis factor α
TUNEL: Terminal deoxynucleotidyl transferase dUTP nick end labeling
